# Case Report: Azathioprine-induced Sweet’s syndrome with associated myocardial infarction

**DOI:** 10.3389/fmed.2025.1548941

**Published:** 2025-05-27

**Authors:** Natalie Commins, Deloshaan Subhaharan, Lauren White

**Affiliations:** ^1^Department of Gastroenterology, Sunshine Coast University Hospital, Birtinya, QLD, Australia; ^2^Department of Gastroenterology, Gold Coast University Hospital, Southport, QLD, Australia

**Keywords:** Sweet’s syndrome, inflammatory bowel disease, azathioprine, myocardial infarction, drug reaction

## Abstract

Sweet’s syndrome (SS) is an acute febrile neutrophilic dermatosis characterized by a rash with tender erythematous plaques or nodules, fevers and leukocytosis. Drug-induced SS is a subtype of SS which is associated with a growing number of medications including azathioprine (AZA). Cardiovascular complications secondary to SS are rare but have been described in case reports and include myocardial infarction and myopericarditis. We report a man in his 60’s with left-sided colonic inflammatory bowel disease (IBD) and perianal disease who developed SS 3 weeks post commencement of AZA. His admission was complicated by an inferolateral ST elevation myocardial infarction occurring concurrently with SS. Clinical improvement occurred with AZA cessation and introduction of intravenous steroids.

## Introduction

Sweet’s syndrome (SS) describes an acute febrile neutrophilic dermatosis which is characterised by a distinctive rash, fevers and leukocytosis (1). The extracutaneous manifestations of SS can affect nearly all organ systems and can cause permanent damage (2). A number of case reports have described cardiovascular complications in the setting of SS, which include myocardial infarction, myocarditis and myopericarditis (3–5). SS has been associated with a number of different medications including those used in the treatment of IBD such as azathioprine (AZA) and anti-TNF agents (6, 7). Classically, AZA-induced SS occurs 1–4 weeks post commencement of the medication (8). We present a severe case of drug-induced SS secondary to AZA complicated by concurrent myocardial infarction at the onset of development of SS symptoms. There was prompt resolution of illness with cessation of AZA and treatment with corticosteroids.

## Case description

**Figure 1:**
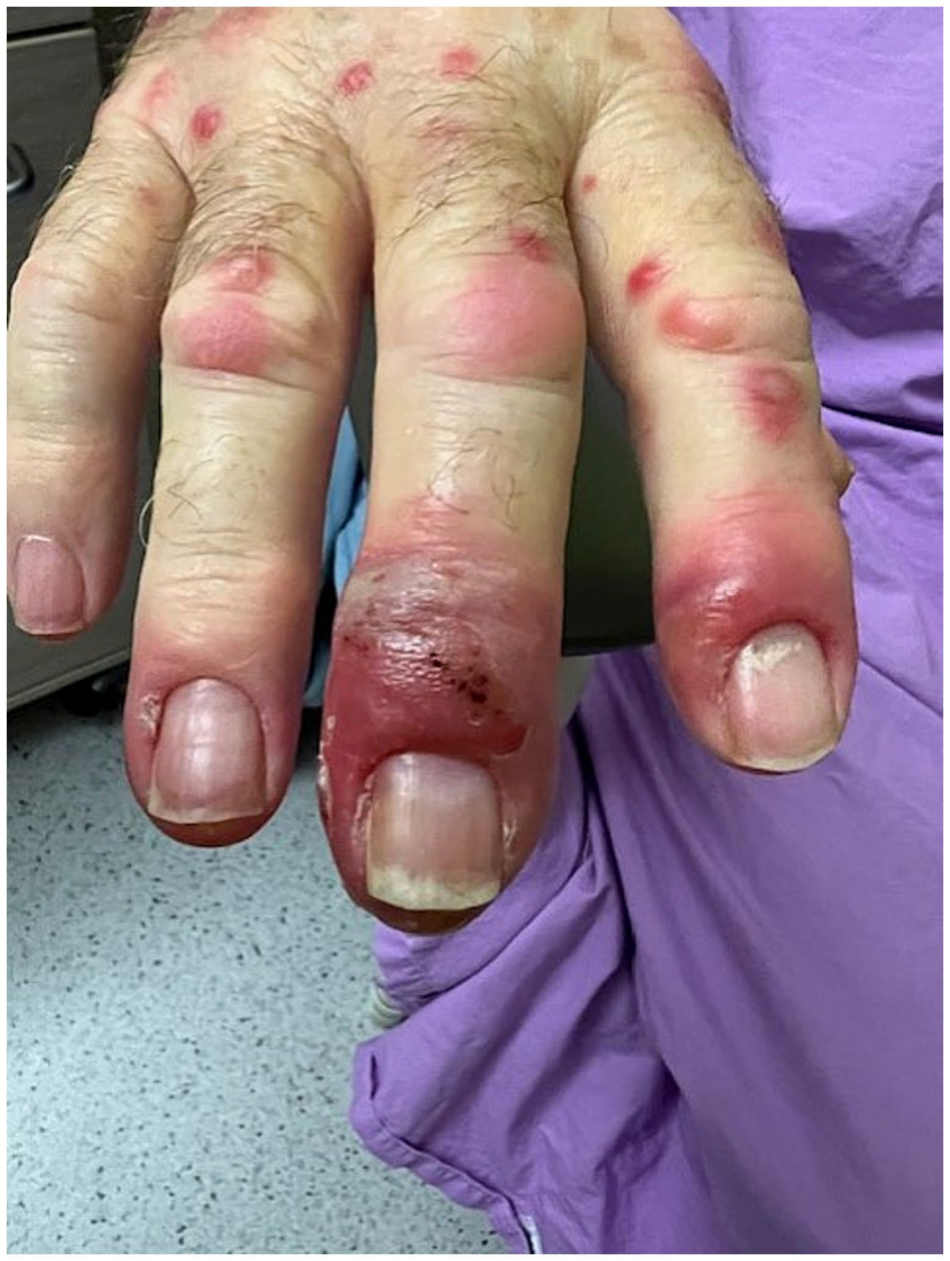
Multiple erythematous edematous papules of the hand with associated plaques, pustules and a deroofed blister over the third digit.

A male in his 60s presented to the emergency department with fevers, tachycardia and ischaemic electrocardiogram changes. He developed infective symptoms 3 days prior to presentation with fevers, rigors, lethargy and anorexia. He had a history of several hours of chest tightness 2 days prior to admission. His past medical history included left-sided colonic IBD with perianal disease which was diagnosed 6 months prior to presentation. He was commenced on infliximab (400 mg IV monthly) 3 months prior and AZA (50 mg daily) 3 weeks prior to presentation. His medical history is remarkable for ankylosing spondylitis (AS), hypertension, hyperlipidaemia, impaired glucose tolerance, gastro-oesophageal reflux disease, mild cognitive impairment and obstructive sleep apnoea. He had a recent CT of his coronary arteries with a coronary calcium score of 900 suggestive of significant coronary artery disease (CAD). His medications at presentation were AZA, infliximab, budesonide foam enemas, pantoprazole, aspirin, ezetimibe and rosuvastatin. He was a non-smoker and had occasional alcohol use. On admission he was noted to have a papular rash over hands and legs. This quickly developed into a erythematous eruption with papules and plaques (Figure 1) with later development of widespread pustules.

## Diagnostic assessment

His laboratory results on admission were remarkable for an elevated white cell count of 18×10^9^/L (reference range 4-11^9^/L) with neutrophilia. His C-reactive protein was elevated at 145 mg/L (reference range <5 mg/L). He had a mild normocytic anaemia with a haemoglobin of 110 g/L (reference range 135-180 g/L). He had a troponin of 29,449 ng/L (reference range <54 ng/L) with associated ST elevation and q waves in inferior leads on electrocardiogram. He had an acute kidney injury with a serum creatinine of 145 μmol/L compared to baseline creatinine of 76 μmol/L (reference range 64-108 μmol/L). Liver function testing revealed an aspartate aminotransferase of 114 U/L (reference range <35 U/L) but otherwise was normal. He returned negative results for hepatitis A, B and C, human immunodeficiency virus, cytomegalovirus, herpes simplex virus, varicella zoster virus, stool PCR for bacterial, viral and parasitic infections, Q fever, syphilis, legionella, *streptococcus pneumoniae*, rickettsia, strongyloides and tuberculosis antigens. Microscopy, sensitivity and culture performed on a swab from a pustule grew no organisms. Blood cultures taken serially through admission were negative. Immune testing was performed with anti-*saccharomyces cerevisiae* IgG elevated at 44.7 U/mL (reference range <20 U/mL) and elevated atypical p-antineutrophil cytoplasmic antibodies of 640CU (reference range <20CU). Antinuclear antibodies, double-stranded DNA, extractable nuclear antigens, complement proteins and anti-glomerular basement membrane antibodies were all within range. His faecal calprotectin was elevated at 410 ug/g (reference range <50 ug/g). His 6-thioguanine and 6-methyl mercaptopurine levels were normal at 146 pmol/8×10^8^ RBC and 386 pmol/8×10^8^ RBCpmol/8×10^8^ RBC, respectively. He underwent a transthoracic echocardiogram which revealed regional wall motion abnormalities in the inferoposterolateral distribution and was diagnosed with an inferolateral myocardial infarction for which he received treatment with fondaparinux. His renal function worsened with a peak creatinine of 404 μmol/L.

**Figure 2:**
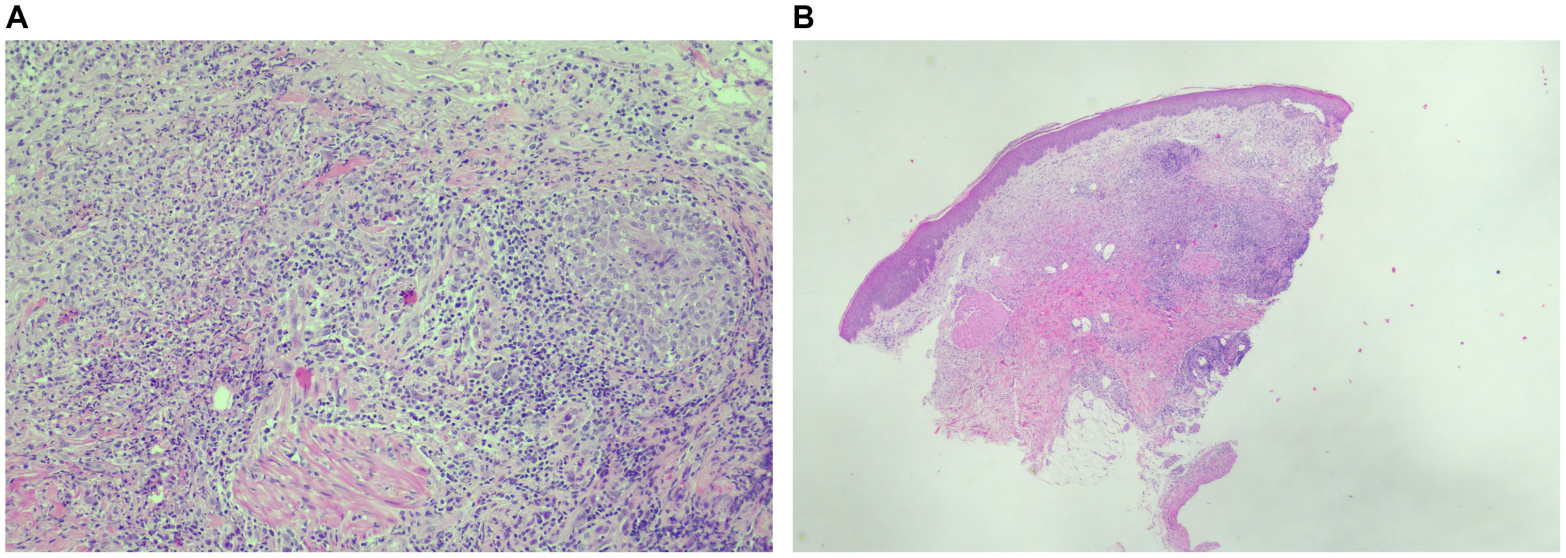
**(A,B)** Punch biopsy of the left forearm demonstrating intradermal dense interstitial and perivascular neutrophilic infiltrate.

Punch biopsies of the forearm were obtained and sent for histopathology (Figures 2A,B). This demonstrated a dense interstitial, perivascular and periadnexal inflammatory infiltrate in the dermis predominantly comprising of neutrophils, histiocytes and lymphocytes without evidence of vasculitis. Direct immunofluorescence from a lesional biopsy was performed which was negative for IgG, IgA and IgM and focal staining for fibrin within the upper dermis. Findings were in keeping with SS.

He was commenced on IV hydrocortisone 50 mg six-hourly for empiric treatment of presumed SS pending biopsy. AZA had already been ceased on admission. Within two days there was rapid resolution of fevers with improving rash. He was transitioned to oral prednisolone on discharge and his renal function improved back to baseline.

Oral prednisolone was weaned thereafter over the next 5 weeks with complete resolution of rash. He received topical betamethasone dipropionate treatment until the rash resolved. Infliximab was not felt to be the causative agent and therefore was continued ongoing for maintenance without recurrence of SS.

## Discussion

SS is a systemic inflammatory syndrome which manifests as a neutrophilic dermatosis that is most commonly idiopathic but also can occur secondary to an underlying disorder (7). It is characterised by fever, erythematous and tender skin lesions, neutrophilia and typical histological findings of a diffuse infiltrate of neutrophils in the upper dermis (9). The three subtypes of SS include classic, drug-induced and malignancy-associated. Classic SS is the most common subtype and includes idiopathic cases as well as those associated with a number of underlying causes such as infection, pregnancy, autoimmune and inflammatory conditions (10).

Drug-induced SS has been attributed to a number of different medications, the most common being granulocyte-colony stimulating factor (G-CSF) (9). A recent review by Heath et al. identified at least 49 drugs implicated in drug-induced SS which included AZA, all-trans retinoic acid (ATRA), hydralazine and bortezomib (10). The association of G-CSF and SS offers some insight into the pathogenesis of SS, as G-CSF plays an important role in innate immune system signaling and is responsible for neutrophil differentiation, maturation and activation (10). SS has also been associated with anti-TNF agents such as infliximab and adalimumab (6, 11, 12). Paradoxically, infliximab and adalimumab are also used as therapeutic agents in the treatment of SS refractory to glucocorticoid therapy (13, 14).

AZA is an immunosuppressant medication which metabolises to cytotoxic thioguanine nucleotides which inhibit purine synthesis and leads to decreased leukocyte proliferation (15). It is used as a therapeutic agent in a wide variety of diseases including inflammatory bowel disease and is one of the more common causes of drug-induced SS. The pathogenesis of AZA-induced SS is not well understood though is theorized to develop as a result of cytokine and mediator release induced by AZA (16). Typically SS occurs within 1–4 weeks after commencement of AZA and systemic involvement is rare (8). A review of 18 cases of AZA-induced SS found the majority of patients had IBD with most cases occurring within 2 weeks of treatment (17).

Diagnostic criteria for classical SS has two major criteria, both of which must be present – sudden onset of tender plaques or nodules, and histology demonstrating neutrophilic infiltrate in the dermis without vasculitis (18). Specific criteria developed for drug-induced SS require these major criteria to be fulfilled with the addition of three further criteria - fever greater than 38°C, a temporal relationship between drug ingestion and symptoms, and resolution of lesions after drug withdrawal or treatment with corticosteroids (19). All criteria were met in this patient. SS is also considered to be an extraintestinal manifestation of IBD and may occur in the setting of active or inactive disease (8). This was considered in our patient however was not felt to be the case given the timeline for recently commenced AZA and prompt resolution of rash and fevers. AS has also been associated with SS however was not felt to be contributory given our patient’s AS was in remission with no recent flares. Other differentials that were considered included vasculitis (which may have biopsy findings similar to SS), drug eruption, erythema nodosum and disseminated HSV.

AZA hypersensitivity syndrome was considered in our patient as it typically occurs within 4 weeks of commencing azathioprine and is dose independent (20). Cutaneous manifestations such as SS, erythema nodosum and small-vessel vasculitis may occur, in addition to a wide range of systemic features such as fevers, diarrhoea, arthralgias and rarely multiorgan involvement (20). Histology demonstrated typical neutrophilic infiltrate in the dermis as opposed to the lymphocytic infiltrates and eosinophils that are more commonly seen in AZA hypersensitivity. The response to steroids tends to be more delayed in AZA hypersensitivity syndrome whereas our patient had swift response to treatment. Neutrophilic dermatosis of the dorsal hands (NDDH) was also considered in our patient and refers to a localized variant of SS limited to the hands (21). Our patient had the typical erythematous nodules and plaques on the extensor surfaces of his hands but had involvement of his legs and trunk which did not fit with NDDH.

The pathogenesis of SS is not well understood but is thought to be secondary to autoinflammation that is driven by innate immune system dysregulation (22). Cardiovascular complications secondary to SS are rare but have been described. Myocardial infarction has been described to occur in the setting of SS (3). Myocarditis and myopericarditis secondary to SS has also been described (4, 23). The mechanism of SS-induced myocardial infarction is not well understood. Elevated inflammatory markers such as IL-6 and TNF-*α* contribute to atherosclerosis and can lead to CAD (5). Our patient was at risk of ischaemic heart disease with a history of CAD and a number of risk factors including hypertension, hypercholesterolaemia, impaired gluclose tolerance and autoimmune inflammatory conditions (IBD and AS). SS was likely the inciting factor in the development of myocardial infarction given the onset of chest pain occurring simultaneously with the development of severe SS. The myocardial infarction in the setting of severe SS may be a consequence of SS or alternatively could have been a Type 2 myocardial infarction (demand related ischaemia) in the setting of significant concurrent illness. The patient did not undergo a coronary angiogram and as such a Type 1 myocardial infarction (coronary artery event such as plaque rupture with thrombus formation) was not excluded though was felt less likely in this circumstance.

Anti-TNF agents such as infliximab and adalimumab can cause drug-induced SS but are also utilized as therapeutic options in treating refractory SS. This dilemma highlights the importance of a clear drug history in evaluating for potential causes of SS, especially in patients with IBD where multiple therapeutic agents are associated with the development of SS and also a flare of IBD itself can cause classical SS. Our patient was commenced on infliximab three months prior to the development of SS and such AZA was felt to be the cause of his disease given onset of symptoms three weeks post commencing AZA and swift resolution of symptoms with withdrawal of the drug. Drug rechallenge with AZA would further strengthen the diagnosis of AZA-induced SS however was not considered in this case as SS was severe and associated with multi-organ dysfunction.

## Data Availability

The original contributions presented in the study are included in the article/Supplementary material, further inquiries can be directed to the corresponding author/s.
